# Correlation between the secondary structure of pre-mRNA introns and the efficiency of splicing in *Saccharomyces cerevisiae*

**DOI:** 10.1186/1471-2164-9-355

**Published:** 2008-07-29

**Authors:** Sanja Rogic, Ben Montpetit, Holger H Hoos, Alan K Mackworth, BF Francis Ouellette, Philip Hieter

**Affiliations:** 1 Department of Computer Science, University of British Columbia, Vancouver, Canada; 2 Center for High-Throughput Biology, University of British Columbia, Vancouver, Canada; 3 Michael Smith Laboratories, University of British Columbia, Vancouver, Canada; 4 Centre for Molecular Medicine and Therapeutics, Vancouver, Canada; 5 Ontario Institute for Cancer Research, Toronto, Canada

## Abstract

**Background:**

Secondary structure interactions within introns have been shown to be essential for efficient splicing of several yeast genes. The nature of these base-pairing interactions and their effect on splicing efficiency were most extensively studied in ribosomal protein gene *RPS17B *(previously known as *RP51B*). It was determined that complementary pairing between two sequence segments located downstream of the 5' splice site and upstream of the branchpoint sequence promotes efficient splicing of the *RPS17B *pre-mRNA, presumably by shortening the branchpoint distance. However, no attempts were made to compute a shortened, 'structural' branchpoint distance and thus the functional relationship between this distance and the splicing efficiency remains unknown.

**Results:**

In this paper we use computational RNA secondary structure prediction to analyze the secondary structure of the *RPS17B *intron. We show that it is necessary to consider suboptimal structure predictions and to compute the structural branchpoint distances in order to explain previously published splicing efficiency results. Our study reveals that there is a tight correlation between this distance and splicing efficiency levels of intron mutants described in the literature. We experimentally test this correlation on additional *RPS17B *mutants and intron mutants within two other yeast genes.

**Conclusion:**

The proposed model of secondary structure requirements for efficient splicing is the first attempt to specify the functional relationship between pre-mRNA secondary structure and splicing. Our findings provide further insights into the role of pre-mRNA secondary structure in gene splicing in yeast and also offer basis for improvement of computational methods for splice site identification and gene-finding.

## Background

Splicing of precursor mRNA is one of the essential cellular processes in eukaryotic organisms. Although this process has been extensively studied since the discovery of splicing three decades ago [1,2], resulting in a thorough understanding of the splicing pathway and identification of the numerous components of the splicing machinery, there are still many unanswered questions. For example, while the ability of pre-mRNA to form intramolecular interactions between short complementary segments in long yeast introns was initially suggested 20 years ago [3], the role of pre-mRNA secondary structure in splicing is not well understood.

Introns in *S. cerevisiae *are known to have bimodal length distribution [4] and can be classified into short and long introns based on their length. The distance between the 5' splice site and the branchpoint sequence, also known as the 'lariat length' or 'branchpoint distance' (we also refer to it as linear branchpoint distance), is tightly correlated with intron length (with a Pearson correlation coefficient of *r *= 0.99 [5]) and can also be used to classify introns into long (5'L) and short (5'S) [3]. It was hypothesized that 5'L introns, for which the branchpoint distance is greater than 200 nt, can fold into secondary structures to optimize the positioning of the 5' splice site and branchpoint sequence to one that is optimal for spliceosome assembly [3]. This hypothesis was confirmed for a limited number of yeast introns by comprehensive biological experiments that demonstrated that the existence of such secondary structure elements is essential for splicing efficiency [6-11]. Structural elements that exhibit a similar effect on splicing efficiency were also found in introns of *Drosophila melanogaster *and related species [12]. Furthermore, in mammalian cells, folding of long intron sequences is facilitated by protein binding and interactions, which presumably shortens the long distance between essential splicing sequences [13].

The nature of the base-pairing interactions within introns and their effect on splicing efficiency were most extensively studied in *S. cerevisiae's *ribosomal protein gene *RPS17B*, previously known as *RP51B *(YDR447C). It was shown that secondary structure interaction between two sequence segments located downstream of the 5' splice site and upstream of the branchpoint sequence promotes efficient splicing of the *RPS17B *pre-mRNA [7]. This interaction was further tested by comprehensive mutational and structure-probing analysis to determine the structure of the stem formed in the wildtype intron and the sensitivity of splicing efficiency to the alterations in this stem [8,9]. These studies demonstrated that complementary pairing between two ends of the *RPS17B *intron, but not necessarily the formation of the described stem, is essential for its efficient splicing *in vitro *and *in vivo*.

While the authors of the previous studies speculated that the function of the complementary pairing is to shorten the branchpoint distance, they did not attempt to determine the secondary structure of the intron and the resulting 'structural' branchpoint distance. Thus a functional relationship between this distance and the splicing efficiency remains unknown.

In this paper we use computational RNA secondary structure prediction to investigate the secondary structures of wildtype and mutant intron sequences within the *S. cerevisiae RPS17B *pre-mRNA. We present a unique algorithm for measuring 'structural' distance between two bases in an RNA secondary structure and use it to compute the distance between the 5' splice site and the branchpoint sequence based on the predicted secondary structure. Our analysis show that there is a tight correlation between structural branchpoint distances and splicing efficiency levels for all mutants examined.

## Results

### Secondary structure of *RPS17B *intron and the efficiency of splicing

The first goal of our study was to determine if the splicing efficiency results previously reported for *RPS17B *intron [8] can be correlated with the computationally predicted secondary structures of wildtype and mutant intron sequences.

In this study the sensitivity of splicing to alterations in the stem formed in the *RPS17B *intron was tested by introducing mutations in the interacting regions designated UB1 (upstream box 1) and DB1 (downstream box 1). The assumption behind the mutant design was that any mutation within the stem would disrupt it and change the intron secondary structure in such a way that the resulting structural branchpoint distance (*d*_*s*_) would be greater than for the wildtype intron. The authors created 9 mutant introns within the *RPS17B *gene: *3mUB1 *(3 nt mutation), *4mUB1 *(4 nt), *5mUB1 *(5 nt), *6mUB1 *(6 nt) and *8mUB1 *(8 nt), where mutations fall in the UB1 region; *3mDB1 *(3 nt) and *5mDB1 *(5 nt), where mutations fall in the DB1 region and are designed to restore the base-pairing disrupted by the mutations in the *3mUB1 *and *5mUB1*, respectively; and *3mUB1_3mDB1 *and *5mUB1_5mDB1*, which are double mutants. All of the single mutants are expected to disrupt the secondary structure, while the double mutants are predicted to restore it. The *RPS17B *intron was inserted into the coding region of the copper resistance gene (*CUP1*), which served as a reporter gene. Thus, yeast cells grown on copper containing medium will be viable only if the intron-containing *Cup1 *mRNA is spliced. The results of this assay suggested that for all single mutants except *8mUB1*, splicing was reduced. Surprisingly, *8mUB1 *had a similar growth rate on copper media as the wildtype intron suggesting that splicing was as efficient. Out of two double mutants, *5mUB1_5mDB1 *was able to partially rescue copper resistance, while *3mUB1_3mDB1 *did not. The authors hypothesized that these unexpected results were the result of some secondary structure rearrangements; however, the secondary structure of the mutants *8mUB1 *and *3mUB1_3mDB1 *was not explored.

In order to investigate if the differences in the splicing efficiency levels are due to the differences in secondary structures, we computed the minimum free energy (MFE) structures of the introns using mfold [14,15], one of the most frequently used RNA secondary structure prediction tools. The comparative RNA secondary structure prediction, which is considered more reliable, requires a certain number of orthologous sequences which were available only for the wildtype *RPS17B *intron and not for the mutants created in [8].

According to the mfold MFE predictions, the introduced mutations have the desired effect of disrupting the stem in all single mutants, but the compensatory mutations fail to restore it in two double mutants. Focusing on the positioning between the donor site and the branchpoint sequence, we compared the part of the structure that contains these two sites across all the mutants. The specified structural domain was almost identical for the *3mUB1*, *5mUB1*, *8mUB1*, *3mDB1*, *3mUB1_3mDB1 *and *5mUB1_5mDB1 *mutants, some of which have very different splicing efficiency levels (see Additional file 1). Moreover, the full secondary structures of the *3mUB1 *and *3mUB1_3mDB1 *mutants were almost identical with only three base-pairs difference, while the copper resistance experiment suggested significant differences in splicing efficiency. Therefore, it appears that differences in the splicing efficiency of Libri et al.'s [8] mutants cannot be attributed to differences in the computed MFE secondary structures of introns.

However, considering only a single, minimum free energy secondary structure prediction of an intron might not be the appropriate approach. While functional, non-coding RNAs, such as tRNAs and rRNAs, have a strong evolutionary pressure to maintain their unique, functional structure, it is believed that mRNAs, whose primary role is to carry the protein coding information to the translation apparatus, do not have functional constraints on their global structure. Thus, instead of always folding into unique MFE structure, it is likely that mRNAs exist in a population of structures [16-18]. Another reason for considering suboptimal structures, especially when using computational prediction methods, is that RNA secondary structure prediction algorithms have limited accuracy and sometimes the correct structure is buried among the suboptimal predictions with free energies very close to the MFE [15,19,20].

#### Structural branchpoint distances of suboptimal secondary structures and the efficiency of splicing

Based on these considerations, we modified our approach to include not only the optimal, i.e., MFE structure, but also near-optimal predictions whose free energies are within 5% of the optimum. There is an exponential relationship between the free energy of a structure and its probability in the ensemble of all possible structures for a given sequence. The probability of a structure *S*_*i *_in the Boltzmann ensemble of all possible structures (*S*_1_, *S*_2_,...) for a given RNA sequence is given by:

(1) $$P(S_{i})=\frac{e^{-\Delta G(S_{i})/RT}}{Q}$$

where Δ*G*(*S*_*i*_) is the free energy of structure *S*_*i*_, *Q *= Σ_*S*_* e*^-Δ*G*(*S*)/*RT *^the partition function for all possible secondary structures for the given sequence, *R *is the physical gas constant, and *T *is the temperature. The probability of a secondary structure is also called the Boltzmann weight of that structure.

From the equation we can see that the lower the free energy of a structure the higher its probability, thus, the predictions within 5% from the MFE also represent the most probable structures for a given sequence, with the MFE prediction being the one with the highest probability.

We used RNAsubopt algorithm [20] to sample 1000 suboptimal structures within 5% of the MFE for each considered intron. RNAsubopt first calculates all suboptimal structures within a user defined energy range and then produces a random sample of structures, drawn with probabilities equal to their Boltzmann weights. Therefore, RNAsubopt computes a representative sample of the secondary structure space within 5% of the MFE.

Since the pair-wise structure comparison and distance estimation approach that we used for MFE structure predictions were not applicable to large number of structures we had to devise a new way to quantify the structural distance between the donor site and the branchpoint sequence. We designed an algorithm that converts an RNA secondary structure into a graph and then applies a shortest-path algorithm from graph theory to compute the shortest distance between two bases in the secondary structure. To the best of our knowledge this is the first algorithm for structural distance computation. More details are given in Materials and Methods.

**Figure 1:**
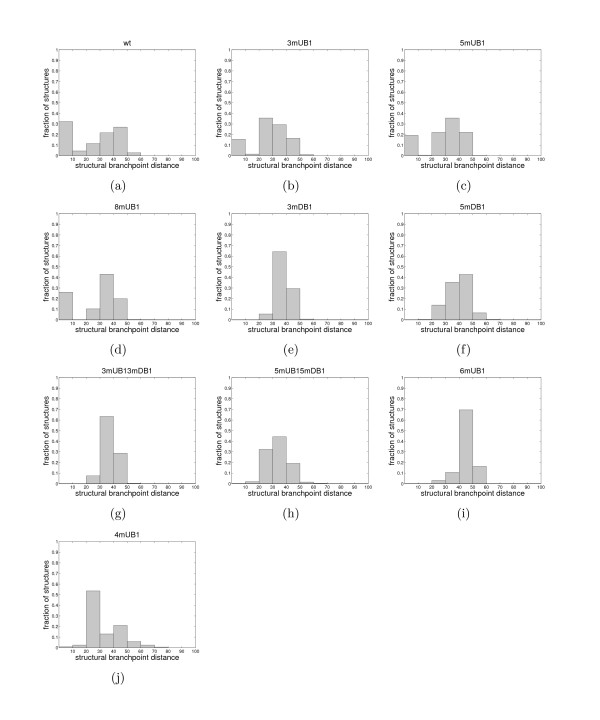
Distribution histograms of structural branchpoint distances for (a) wt, (b) *3mUB1*, (c) *5mUB1*, (d) *8mUB1*, (e) *3mDB1*, (f) *5mDB1*, (g) *3mUB1_3mDB1*, (h) *5mUB1_5mDB1*, (i) *6mUB1*, and (j) *4mUB1 *introns.

For each secondary structure prediction, we computed the exact distance between the donor site and the branchpoint sequences (*d*_*s*_) using the shortest-path algorithm. The average structural branchpoint distances are given in Table 1. We assigned descriptive splicing efficiency labels based on the gel images in Figure 2A in [8]. The distributions of computed structural branchpoint distances for each of the *RPS17B *mutants are given in Figure 1.

**Table 1:** Average structural branchpoint distances for the wildtype (wt) *RPS17B *intron and Libri et al.'s [8] intron mutants.

**mutant**	**average *d*_*s*_**	**splicing efficiency**
**wt**	26.67	efficient (1)
***3mUB1***	27.67	slightly reduced (2)
***5mUB1***	28.42	slightly reduced (2)
***8mUB1***	27.94	efficient (1)
***3mDB1***	37.55	inhibited (4)
***5mDB1***	39.19	inhibited(4)
***3mUB1_3mDB1***	37.44	inhibited (4)
***5mUB1_5mDB1***	33.81	slightly reduced (2)
***6mUB1***	46.31	inhibited (4)
***4mUB1***	32.08	reduced (3)

Levels of splicing efficiency were approximated from the gel images in Figure 2A in [8]. The numbers within parentheses correspond to numerical values assigned to descriptive splicing efficiency labels.

**Figure 2:**
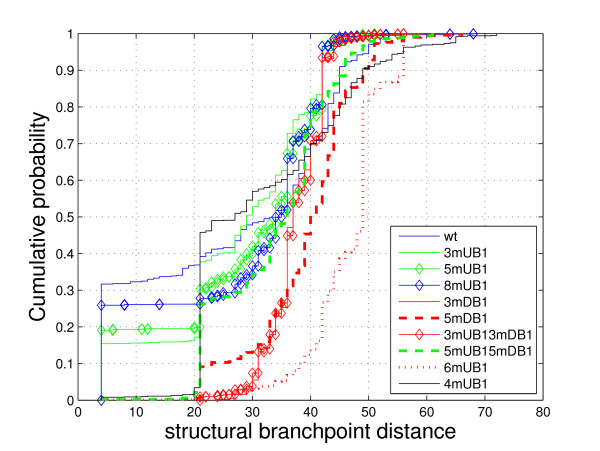
Cumulative distributions of structural branchpoint distances for all Libri et al.'s [8] intron mutants.

These results suggest an interesting correlation between the average structural branchpoint distance and the splicing efficiency levels: sequences that are more efficiently spliced (wildtype, *3mUB1*, *5mUB1*, *8mUB1*, *5mUB1_5mDB1*, and *4mUB1*) have lower values for the average distance than those that are poorly spliced. After assigning numerical values to the descriptive splicing efficiency labels (efficient = 1, slightly reduced = 2, reduced = 3 and inhibited = 4) we obtain a Pearson correlation coefficient of 0.87.

The histograms in Figure 1 offer further insights into the relationship between structural branchpoint distances of introns and their efficiency of splicing; introns that are spliced efficiently or with slightly reduced efficiency have large frequency of suboptimal structures with *d*_*s *_< 10. Mutant *5mUB1_5mDB1*, which does not have this prominent peak in its distribution histogram and mutant *4mUB1*, which has reduced splicing efficiency, but not completely inhibited, still have higher frequency of structures with *d*_*s *_< 20 than the remaining, poorly spliced mutants. The correlation coefficient between splicing efficiency level and the proportion of structures with *d*_*s *_< 20 is 0.85.

Finally, the cumulative distribution plot of structural branchpoint distances for all mutants, where lines are labeled according to the splicing efficiency levels (efficient – blue, slightly reduced – green, reduced – black and inhibited – red) shows a clear separation of spliced and unspliced mutants (Figure 2).

Upon closer inspection we noticed that most of the structures with *d*_*s *_< 10 have *d*_*s *_= 4. Analysis of the secondary structures of these sequences reveals that this distance corresponds to a structural conformation where the donor and branchpoint sequences have two base-pairing interactions between them (see Section 2.1.3). The observed base-pairing interactions are not necessarily inconsistent with established models of the splicing process, according to which spliceosomal snRNAs interact with the donor site and the branchpoint sequence, since the base-pairing can be easily disrupted after the splicing factors have been aligned properly.

#### Structural branchpoint distances and the efficiency of splicing for other published *RPS17B *mutants

In order to test the generality of the observed correlation between splicing efficiency levels and structural branchpoint distances we also analyzed the *RPS17B *intron mutants described in [9]. These are *mut-UB1i*, which has an inverted UB1 sequence; *mut-DB1i*, which has an inverted DB1 sequence; *mut*-*UB1iDB1i*, which has both UB1 and DB1 sequences inverted to make them complementary to each other; *mut-5*, which reduces the consecutive pairing region to 5 base-pairs, *mut-12*; which improves pairing to 12 consecutive base-pairs (eliminating one one-nucleotide bulge); and *mut-18*, which extends pairing to 18 consecutive base-pairs (eliminating all three bulges in the pairing region). The authors compared splicing efficiency of the wildtype and mutant introns by analyzing the formation of spliceosomal complexes. Based on their gel images, we assigned descriptive and numerical splicing efficiency labels to the tested sequences (see Table 2). The average structural branchpoint distances of 1000 suboptimal structures sampled from within 5% of the MFE for each mutant are given in Table 2.

**Table 2:** Average structural branchpoint distances for the wildtype (wt) *RPS17B *intron and Charpentier and Rosbash's [9] intron mutants.

**mutant**	**average *d*_*s*_**	**splicing efficiency**
**wt**	26.67	normal (2)
***mut-UB1i***	42.51	reduced (3)
***mut-DB1i***	35.95	reduced (3)
***mut-UB1iDB1i***	26.39	improved (1)
***mut-5***	32.14	reduced (3)
***mut-12***	24.82	improved (1)
***mut-18***	25.30	improved (1)

**Figure 3:**
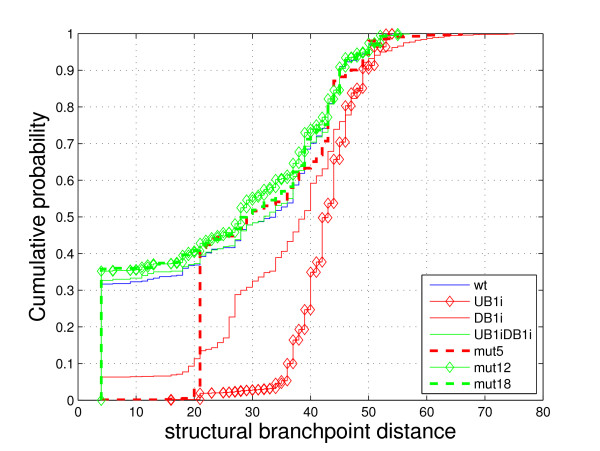
Cumulative distributions of structural branchpoint distances for all Charpentier and Rosbash's [9] intron mutants.

We inferred levels of splicing efficiency based on Figures 2 and 3 and Table 1 in [9]. The numbers within parentheses correspond to numerical values assigned to descriptive splicing efficiency labels.

The branchpoint distance results for these mutants are similar to those of Libri et al.'s [8] mutants; the average structural branchpoint distances are lower for the sequences that are efficiently spliced (wildtype, *mut-UB1iDB1i*, *mut-12*, and *mut-18*). After assigning numerical values to the descriptive splicing efficiency labels (improved = 1, normal = 2 and reduced = 3), we obtain the correlation coefficient as 0.85. This, again, corresponds to the ability of these sequences to fold in such a way as to bring the donor site and the branchpoint sequences close to each other; each of the efficiently spliced sequences has a large fraction of predicted secondary structures for which *d*_*s *_< 10 (Figure 3 and Additional file 2). The mutants that show reduced splicing have very few of these structures (0.02% for *mut-UB1i *and 0.33% for *mut-5*), except for *mut-DB1i*, which has 11.3% of structures with *d*_*s *_< 10. However, this is still significantly lower than for the efficiently spliced mutants. Again, the cumulative distribution plot clearly separates mutants based on their splicing efficiency (Figure 3).

#### Base-pairing probabilities of the RPS17B intron and the efficiency of splicing

**Figure 4:**
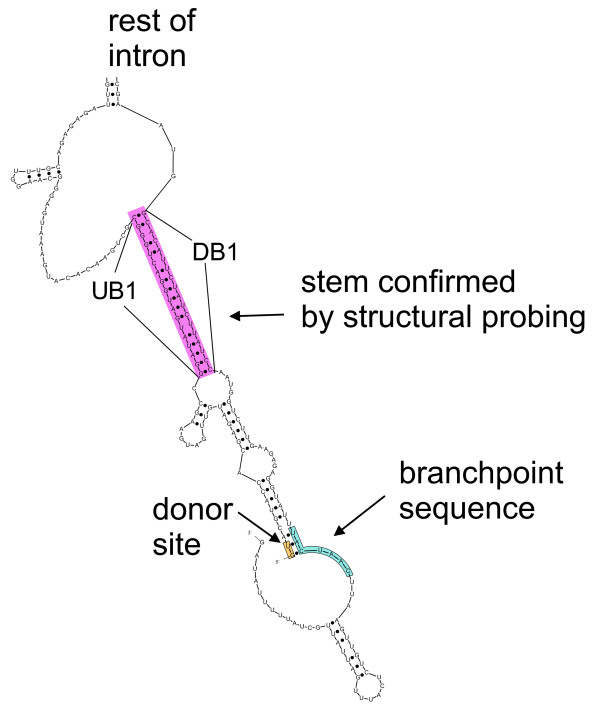
A part of the wildtype *RPS17B *intron secondary structure that shows base-pairing between the donor site and the branchpoint sequence. The highlighted stem is the same as the one identified in [9] using experimental structure probing.

The branchpoint distance analysis of *S. cerevisiae's *RPS17B intron suggests that the ability to form highly probable secondary structures (within 5% of the MFE) with short distance between the donor site and the branchpoint sequence seems to be required for efficient splicing of the intron. The short structural branchpoint distance for the *RPS17B *intron results from two base-pair interactions: between the first intron base (G) and the third base of the branchpoint sequence (C); and between the second base in the intron (U) and the second base of the branchpoint sequence (A) (see Figure 4). It is possible to compute the probability of these base-pairing interactions directly using a dynamic programming algorithm that computes the partition function [21]. The base-pair probability reflects a sum of all probability-weighted structures in which the chosen base-pair occurs. Thus, these base-pairing probabilities also take into account the structures that were not within 5% from the MFE, eliminating the necessity to chose an arbitrary percent suboptimality value. The base-pair probabilities can be computed using RNAfold [22], another frequently used program for RNA secondary structure prediction.

The base-pair probability values for the wildtype *RPS17B *intron and all of Libri et al.'s [8] mutants are given in Table 3. The probability values for the two base-pairs (G-C and U-A) are identical up to second decimal place for each intron sequence and that is why only one number is shown in the table. It can be observed that all of the efficiently spliced sequences have higher base-pair probabilities than the poorly spliced sequences (*r *= -0.92). The correlation is not strictly linear since, for example, the mutant sequence *8mUB1 *has almost the same base-pair probability value as *3mUB1 *and *5mUB1*, although it is more efficiently spliced than these two. Similarly, the double mutant *5mUB1_5mDB1 *is more efficiently spliced than *4mUB1*, but this is not reflected in the base-pair probability values.

**Table 3:** Base-pairing probabilities of contact conformation (Figure 4) for the wildtype (wt) *RPS17B *intron and Libri et al.'s [8] intron mutants.

**mutant**	**base-pairing probability**	**splicing efficiency**
**wt**	0.40	efficient
***3mUB1***	0.33	slightly reduced
***5mUB1***	0.31	slightly reduced
***8mUB1***	0.34	efficient
***3mDB1***	0.01	inhibited
***5mDB1***	< 0.01	inhibited
***3mUB1_3mDB1***	0.01	inhibited
***5mUB1_5mDB1***	0.11	slightly reduced
***6mUB1***	0.05	inhibited
***4mUB1***	0.18	reduced

For Charpentier and Rosbash's mutants, the base-pair probabilities are also higher for the sequences that are more efficiently spliced (Table 4): all of the sequences that are efficiently spliced (wildtype, *mut-UB1iDB1i*, *mut-12*, and *mut-18*) have base-pair probabilities of 0.40, while the other sequences have lower values (*r *= -0.85).

**Table 4:** Base-pairing probabilities of contact conformation for the wildtype (wt) *RPS17B *intron and Charpentier and Rosbash's [9] intron mutants.

**mutant**	**base-pairing probability**	**splicing efficiency**
**wt**	0.40	normal
***mut-UB1i***	0.04	reduced
***mut-DB1i***	0.25	reduced
***mut-UB1iDB1i***	0.40	improved
***mut-5***	0.04	reduced
***mut-12***	0.40	improved
***mut-18***	0.40	improved

Overall, based on the results for Libri et al.'s [8] and Charpentier and Rosbash's [9] mutants it seems that, at least for *RPS17B *intron, base-pair probabilities for the two base-pairs formed between the first two bases of the intron and the second and third base of the branchpoint sequence are good indicators of splicing efficiency. We will see in the following sections that this is not a general requirement for all genes. Taken together with the observed correlation between the splicing efficiency levels and structural branchpoint distances the results are consistent with the following hypothesis: the existence of highly probable secondary structures that have short branchpoint distance is required for efficient splicing of yeast introns.

### Experimental testing of the hypothesis

In order to test the validity of the proposed hypothesis, we designed and functionally tested *in vivo *a series of *RPS17B *intron mutants. To assay the effect of these mutations on splicing we opted to introduce the mutated intron sequences at their endogenous locus, instead of within the *CUP1 *gene as was previously done [8,9]. This allows us to analyze the splicing of this intron within its normal context of flanking DNA sequences. We estimated the splicing efficiency directly from protein expression levels, which were quantified using a fluorescence imaging system.

**Figure 5:**
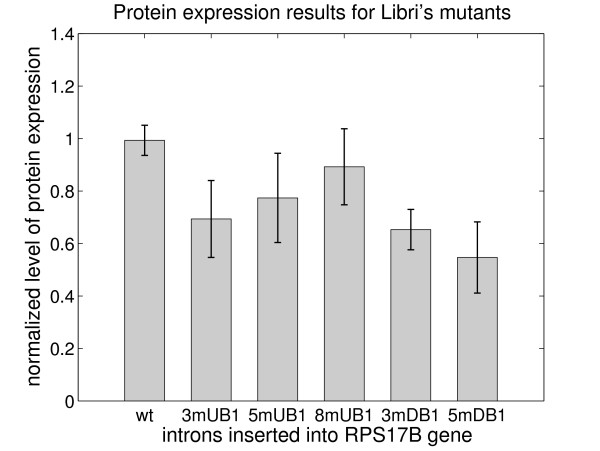
Protein expression levels for the *RPS17B *gene containing some of Libri et al.'s [8] mutant introns. Expression levels are normalized with respect to the internal loading control and plotted as a fraction of the wildtype expression level. Shaded boxes represent the mean value for several different samples and error bars represent +/- 1 standard deviation for these samples. The error bar for the wildtype intron comes from the comparison of two different wildtype samples.

Using protein expression as a measurement of splicing efficiency requires that: 1) the level of protein abundance is proportional to the mRNA abundance (for a given gene) in the cell and, 2) the abundance of mRNA in the cell reflects any change in splicing efficiency. To demonstrate that *RPS17B *follows these general rules, we analyzed a number of Libri et al.'s [8] mutants that have previously documented changes in mRNA levels for their protein expression levels. The sequences tested were the wildtype *RPS17B *intron, and the *5mUB1*, *3mUB1*, *8mUB1*, *5mDB1*, and *3mDB1 *mutated introns. The levels of protein expression, as shown in Figure 5, are proportional to the levels of copper-resistance in the copper growth assay in [8]. Moreover, our approach is able to provide a quantifiable measure for mutants such as *3mDB1 *and *5mDB1*, which did not support any growth in the copper growth assay. Thus, using changes in protein expression levels in the context of different intron sequences to assay the effects of mutations on splicing efficiency is a valid approach.

#### New *RPS17B *intron mutants

We designed 8 new *RPS17B *intron mutants for the purpose of testing our current model of correlation between intronic pre-mRNA secondary structure and splicing efficiency. The most important structural characteristic used for mutant design was structural branchpoint distance (*d*_*s*_) of its MFE and suboptimal structures. Four mutants that are predicted to splice efficiently were designed to have multiple suboptimal structures with contact conformation (Figure 4) and short average structural branchpoint distance (these mutants are labeled with letter 'S', which stands for short *d*_*s*_). The only exception is mutant *rps17b-S2*, which does not have any suboptimal structures with contact conformation, but still exhibits a short structural branchpoint distance (most of the suboptimal predictions have *d*_*s *_= 10). This mutant was designed to test whether contact conformation, rather than the resulting short structural branchpoint distance, is important for splicing. Four mutants that are predicted to have reduced splicing were designed not to have any structures with contact conformation or otherwise short structural branchpoint distances (these mutants are labeled with letter 'L', which stands for long *d*_*s*_).

The mutant design was based on mfold predictions, while RNAsubopt predictions where used post-experimentally to analyze the results. Mfold also samples the suboptimal space of secondary structures, however it does not compute all possible structures and the sample is much smaller. Although the distribution of *d*_*s *_computed based on structure predictions by mfold is similar to the one based on RNAsubopt predictions, the average distances for RNAsubopt predictions are not as distinct between 'S' and 'L' mutants as ones based on mfold predictions.

Table 5 shows average *d*_*s *_for newly designed mutants based on RNAsubopt predictions and base-pair probabilities computed by RNAfold. The analogous table based on mfold suboptimal predictions, which was used in the design process is given in Additional file 3.

**Table 5:** Characteristics of newly designed *RPS17B *mutants.

**mutant**	**avg(*d*_*s*_)**	**bp prob**
**wt**	26.67	0.40
***rps17b-L1***	43.63	0.0
***rps17b-L2***	41.11	0.0
***rps17b-L3***	34.05	0.21
***rps17b-L4***	32.98	0.04
***rps17b-S1***	24.55	0.40
***rps17b-S2***	29.62	0.03
***rps17b-S3***	12.65	0.80
***rps17b-S4***	9.27	0.70

***avg*(*d*_*s*_) **– average structural branchpoint distances of 1000 suboptimal structures predicted by RNAsubopt; **bp prob **– base-pairing probability of interaction between the donor site and the branchpoint sequence based on the partition function.

**Figure 6:**
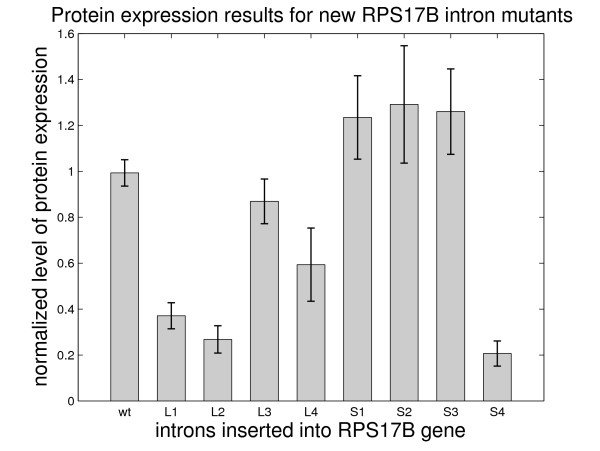
Protein expression results for the *RPS17B *gene containing the newly designed mutant introns.

As seen in Figure 6, mutants *rps17b-L1*, *rps17b-L2 *and *rps17b-L4 *have reduced protein expression levels when compared to the wildtype as expected. Mutant *rps17b-L3 *has reduced splicing efficiency but not as much as the other three mutants with long structural branchpoint distances. As previously explained, this mutant was designed to have reduced splicing based on suboptimal predictions by mfold, which failed to predict any structures with *d*_*s *_< 10. However, RNAsubopt, which does a more rigorous sampling of the suboptimal space, detected a small fraction of suboptimal structures that have *d*_*s *_< 10 (see Additional file 4). This is in agreement with the relatively high probability of base-pairing interaction between the donor site and the branchpoint sequence (0.21).

Mutants *rps17b-S1*, *rps17b-S2*, and *rps17b-S3 *are all spliced efficiently, as predicted. The efficient splicing of mutant *rps17b-S2*, which has short structural branchpoint distance (*d*_*s *_= 10) without contact conformation in many of the predicted structures, suggests that a specific structural arrangement between the donor site and the branchpoint sequence is not required for efficient splicing. Mutant *rps17b-S4 *shows reduced levels of protein abundance, which is in disagreement with our prediction. The mutated sequence for this mutant has the same location as the mutated sequence for the mutant *rps17b-S3*, which is efficiently spliced, thus we can exclude the possibility that the discrepancy in splicing is sequence-based. A possible explanation for this phenomenon may be the existence of a very thermodynamically stable stem (with free energy Δ*G *= -36.6 kcal/mol) that holds the 5' splice site and the branchpoint together (analogous stems in wildtype introns have much higher free energy, see Section 2.3). This stem may be too stable to be disrupted, which might prevent the spliceosome to bind to the splice signals [8]. Overall, the results on the new *RPS17B *intron mutants are consistent with the proposed model of the role of intronic secondary structure in gene splicing in yeast.

#### Selecting additional genes for experimental validation

To further validate our hypothesis regarding the role of intron secondary structure in splicing, we selected additional yeast, intron-containing genes to test our model. The selection criteria were: the linear distance (number of nucleotides) between the donor site and the branchpoint sequence is greater than 200 nt (5'L introns); the intron does not contain an snRNA gene; the gene is not essential (i.e., cells are viable if the gene is mutated or deleted); and the protein product has relatively high abundance in the cell, is amenable to c-terminal tagging, and has molecular weight between 20–120 kDa (to facilitate manipulation).

From our initial dataset of 98 yeast genes that contain 5'L introns (see Materials and Methods), 18 genes matched the selection criteria (17 of these were ribosomal protein genes). We selected two of these for the experiments: the ribosomal protein gene *RPS6B *(YBR181C) and the amino-peptidase gene *APE2 *(YKL157W).

The *RPS6B *gene contains one intron of length 352 nt, with a linear branchpoint distance (the distance between the 5' splice site and the branchpoint sequence) of *d *= 329 nt. The computed structural branchpoint distance (*d*_*s*_) is 18 for the MFE and all the suboptimal computationally predicted secondary structures within 5% of the MFE. Thus for this intron, unlike for the *RPS17B *intron, the donor and branchpoint sequences are not base-paired.

The *APE2 *gene contains one intron of length 383 nt, with a linear branchpoint distance of *d *= 327 nt. One of the suboptimal structures within 5% of the MFE has a structural branchpoint distance of 6 and the others have greater distances. In the suboptimal prediction that has *d*_*s *_= 6 there is no base-pairing interactions between the donor and branchpoint sequences.

#### *RPS6B *intron mutants

We designed intron mutants for the *RPS6B *gene in a similar manner as for the *RPS17B *gene: the mutants that are supposed to have efficient splicing were designed to have similar structural branchpoint distances as the wildtype intron, and the mutants that are supposed to have reduced splicing were designed to have longer distances (see Additional file 5). Table 6 shows average structural branchpoint distances for a sample of 1000 suboptimal predictions within 5% of the MFE and the probability of short branchpoint distance derived form the base-pairing probabilities. The reported probability is the highest base-pair probability between the first donor nucleotide and any nucleotide within 20 bases away from the branchpoint adenosine. This guarantees that the branchpoint distance in a secondary structure that contains that base-pair will be no longer than 20.

**Table 6:** Characteristics of newly designed *RPS6B *mutants.

**mutant**	**avg(*d*_*s*_)**	**bp prob**
**wt**	18.06	0.84
***rps6b-L1***	36.74	0
***rps6b-S1***	19.08	0.65
***rps6b-S2***	18.04	0.84
***rps6b-S3***	18.04	0.83
***rps6b-S4***	18.09	0.84
***rps6b-S5***	22.00	0

***avg*(*d*_*s*_) **– average structural branchpoint distance; **bp prob **– probability of ***d*_*s *_< 20**.

**Figure 7:**
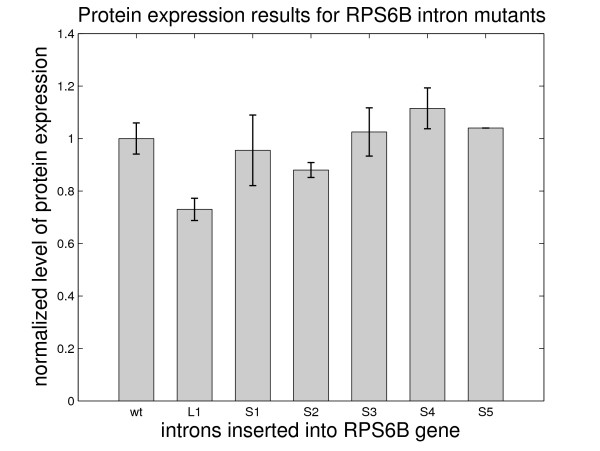
Protein expression results for the *RPS6B *gene containing the newly designed mutant introns.

From Figure 7 we can see that all of the 'S' mutants, which have structural branchpoint distances similar to the wildtype intron, are expressed at levels similar to the wildtype. Mutant *rps6b-L1*, which has *avg*(*d*_*s*_) = 40 shows a reduction in splicing efficiency. The probability of *d*_*s *_< 20 also correlates well with the protein expression data except for mutant *rps6b-S5 *for which *d*_*s *_> 20 for all suboptimal predictions. Thus, for the *RPS6B *gene, structural branchpoint distances slightly longer than 20 seem to be still optimal for splicing. To summarize, the protein expression data for the *RPS6B *gene containing designed intron mutants are compatible with our proposed model of splicing efficiency dependence on the structural branchpoint distance.

#### *APE2 *intron mutants

Using the same selection criteria as before, we designed six *APE2 *intron mutants. The values for average *d*_*s *_and the probabilities of structural branchpoint distance shorter than 20 are given in Table 7, and the histograms of structural branchpoint distance distributions are given in Additional file 6.

**Table 7:** Characteristics of newly designed *APE2 *mutants.

**mutant**	**avg(*d*_*s*_)**	**bp prob**
**wt**	27.90	0.37
***ape2-L1***	75.73	0
***ape2-L2***	69.68	0
***ape2-S1***	8.93	0.82
***ape2-S2***	23.33	0.50
***ape2-S3***	24.60	0.45
***ape2-S4***	25.14	0.42
***ape2-S5***	4.10	0.99

***avg*(*d*_*s*_) **– average structural branchpoint distance; **bp prob **– probability of **d_*s *_< 20**.

**Figure 8:**
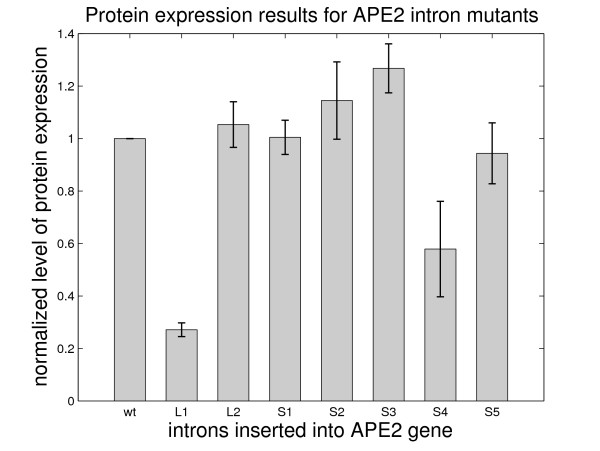
Protein expression results for the *APE2 *gene containing the newly designed mutant introns. Protein expression level is normalized with respect to wildtype expression level. Shaded boxes represent the mean value for several different samples and error bars represent +/- 1 standard deviation for these samples.

The experimental results are consistent with our prediction for five out of seven mutants: mutants *ape2-S1*, *ape2-S2*, *ape2-S3 *and *ape2-S5 *all have a level of protein abundance similar to the wildtype (Figure 8) and mutant *ape2-L1 *shows significantly reduced expression as expected. Mutant *ape2-L2*, which was expected to have reduced protein abundance as a consequence of reduced splicing efficiency, is expressed at the same level as the wildtype. Also, mutant *ape2-S4 *has reduced splicing despite the fact that it has a similar distribution of structural branchpoint distances as the wildtype intron. Since this mutant has the mutation at the same location as *ape2-L1 *(see Materials and Methods), it is possible that the intron segment that we mutated was important for splicing (e.g., contained a splicing enhancer). Overall, the results for *APE2 *mutants support our hypothesis of the role of structural branchpoint distance in gene splicing.

### Shortening of branchpoint distances by zipper stems

**Figure 9:**
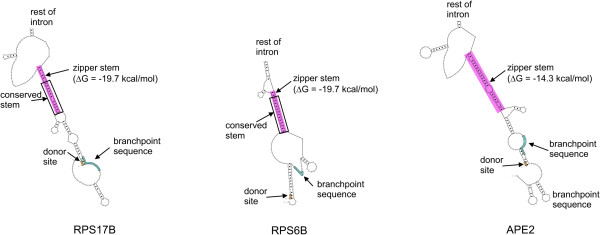
Portions of the *RPS17B*, *RPS6B *and *APE2 *introns containing computationally identified zipper stems. The free energy values (Δ*G*) for the shaded zipper stem are given in parentheses. Stems conserved between *Saccharomyces sensu stricto *group are also labeled.

The splicing efficiency study of *RPS17B*, *RPS6B *and *APE2 *genes containing wildtype and mutant introns supports our hypothesis that short structural branchpoint distances are required for efficient splicing. Although these distances are computed in the context of the secondary structure of the entire intron, our hypothesis is still consistent with the original hypothesis [3] that attributes the shortening of a long branchpoint distance to a single stem. Such stems, which we will refer to as 'zipper' stems, since they 'zip' the intron, are probably essential for achieving a short structural branchpoint distance. If we analyze the computed secondary structures of the *RPS17B*, *RPS6B *and *APE2 *wildtype introns we can easily identify stable stems whose 3' and 5' constituents are close to the donor site and the branchpoint sequence (Figure 9). The zipper stem labeled in the *RPS17B *intron is the same as the one identified in [9] using experimental structure probing.

To further test the functional importance of the identified zipper stems we performed comparative structure analysis using several closely related yeast species (*S. paradoxus*, *S. mikatae*, and *S. bayanus*, as well as *S. cerevisiae*, all belonging to the *Saccharomyces sensu stricto *group). We used multiple sequence alignments to extract the orthologous intron sequences for our three genes [23,24]. Both *RPS17B *and *RPS6B *intron alignments contain three *sensu stricto *sequences. The multiple sequence alignment for *APE2 *contains all four sequences; however, these are not intronic sequences but sequences from the exon 2 of the *APE2 *gene. This error is due to the old *S. cerevisiae *annotation which mapped two genes to the location of the current *APE2 *gene [25].

We computed the consensus structure of *RPS17B *and *RPS6B *introns using Alifold [26]. The previously indicated zipper stems were found in the consensus structures for both genes (Figure 9), thus suggesting evolutionary conservation of these structural elements.

## Discussion

The hypothesis that secondary structure interactions within yeast introns are needed for efficient splicing was proposed two decades ago [3]. Since then, experimental evidence in support of this hypothesis was found for several of *S. cerevisiae's *introns [6-11]. These studies identified complementary segments located downstream of the donor site and upstream of the branchpoint sequence whose base-pairing interactions are essential for splicing. It is conjectured that the function of the formed stem is to bring the donor site and the branchpoint sequence closer together so that they are in optimal alignment for spliceosome assembly.

In this paper we use computational RNA secondary structure prediction to study structural requirements for efficient splicing in yeast. Our approach considers a representative sample of suboptimal structures with free energies close to the MFE and it also considers the entire secondary structure of an intron, rather than a single stem, both of which are more consistent with the nature of RNA molecules. Furthermore, the approach includes a calculation of the structural branchpoint distance, which is used to quantify the effect of the secondary structure on the distance between the donor site and the branchpoint sequence and can easily be correlated with splicing efficiency measurements. Using this method we were able to identify structural characteristics of the *RPS17B *intron and its mutants that seem to be responsible for their splicing differences. Notably, mutants that are likely to have a short structural branchpoint distance are spliced more efficiently.

Based on our model of structural requirements for efficient splicing we computationally designed intron mutants for three *S. cerevisiae genes*, *RPS17B*, *RPS6B *and *APE2*, and experimentally tested their splicing efficiency. The results were mostly consistent with our model, with a few exceptions (*rps17b-L3*, *rps17b-S4*, *ape2-L1 *and *ape2-S4*) which may be due to some structural characteristics of mutants that are not considered by the current model or some inherent approximations in the model that are discussed below. Some of the intron mutants that were designed to have different structural characteristics and splicing efficiencies have mutations at the same locations (e.g., *rps17b-L3 *and *8mUB1*; *rps17b-S3 *and *3mDB1*; *rps6b-L1 *and *rps6b-S3*). The experimental results that confirm differences in splicing between these pairs of mutants indicate that the secondary structure of a pre-mRNA, rather than the underlying primary sequence, is responsible for differences in splicing.

We also tested our model on the *YRA1 *gene intron, whose splicing efficiency had previously been studied by Preker and Guthrie [27]. The published experimental results were in agreement with our model; the efficiently spliced mutants (Δ*L10 *and Δ*TCC*/*GGA*) had higher base-pair probabilities than the poorly spliced sequences (wildtype intron and mutants Δ*R*/*L10*, *TCC *Δ*L10*, *GGA ΔL10 *and *TCC*+*GGA *Δ*L10*) (data not shown).

Our current model is simplified in the sense that the secondary structure of an intron is computed disregarding its flanking sequences, and the three dimensional branchpoint distance is estimated from secondary structure interactions. However, we believe that folding intronic sequences in isolation is appropriate, partly because of the existence of co-transcriptional splicing, where splicing occurs before the entire pre-mRNA has been synthesized [28-30]. Therefore, the precise part of the pre-mRNA that serves as the splicing substrate is not known. The region upstream of the transcribed intron, which consists of the 5' UTR and the first exon, is also not precisely defined due to the fact that the transcription start sites have not been unambiguously mapped [31]. In addition, 5'UTRs are known to associate with a number of protein factors [32,33] which are likely to have an effect on the structure formation, but these interactions are not currently modelled by computational RNA secondary structure approaches. A preliminary investigation, in which we considered some of the upstream region yielded inconclusive results (data not shown). Thus, we believe that folding only intronic sequences gives us a reasonable approximation of the secondary structure of an intron at the time of the splicing reaction.

The approximation of the three dimensional branchpoint distance using pre-mRNA secondary structure is necessary since there are no reasonably reliable algorithms for predicting RNA tertiary structure. However, it is believed that RNA secondary structure plays a crucial role in tertiary structure formation, since most tertiary interactions are thought to arise after the formation of a stable secondary structure, when the molecule is able to bend around the flexible, single-stranded regions [34,35]. Moreover, the tertiary structure interactions that arise in the later stages of folding are usually too weak to disrupt secondary structure that has already formed. Therefore, we believe that the structural branchpoint distance based on the secondary structure interactions provides a reasonable approximation of the true spatial distance.

## Conclusion

Our computational study offers further insights into the role of pre-mRNA secondary structure in gene splicing in yeast. We show that it is necessary to consider near-optimal structure predictions to be able to detect structural differences between intron mutants that have different splicing efficiencies. We also propose a novel method for quantifying a distance between two bases in an RNA secondary structure and apply this to compute structural branchpoint distances in the studied intron mutants. Positive experimental results on three different yeast genes suggest that our model of structural requirements for efficient splicing can be applied universally to all 5'L yeast introns. Additional laboratory experiments are needed to refine the current model by determining the upper bound of the structural branchpoint distance needed for efficient splicing and acceptable thermodynamic stability of the stems adjacent to splicing signals. Considering that several biological studies indicate that shortening of the branchpoint distance, either by formation of secondary structure or by protein interactions, is important for efficient splicing in *Drosophila melanogaster *and some mammalian species [12,13], it might be possible to extend our model to define structural requirements for efficient splicing in other eukaryotes. Another possible application of our findings is in gene-finding, where structural characteristics of identified long introns can be used to distinguish between real and false positive predictions.

## Methods

### Computational RNA secondary structure prediction

In this work we used four different RNA secondary prediction tools: mfold [14,15], RNAsubopt [20], RNAfold [22] and Alifold [26].

Mfold was used for predicting MFE secondary structures for Libri et al.'s [8] mutants and for predicting suboptimal structures within 5% of the MFE during the mutant design process. Mfold uses dynamic programming to identify the MFE secondary structure and a set of suboptimal structures within a user defined percentage from the MFE for a given RNA sequence. We used both, the web (3.2) and command line (3.0) versions of mfold with default parameters.

RNAsubopt was used to compute a sample of 1000 suboptimal structures within the 5% from the MFE. Unlike mfold, it computes *all *suboptimal secondary structures within a user defined energy range or percentage from the MFE for a given RNA sequence. It can also draw a random sample of the computed suboptimal structures using their Boltzmann weights. We used the command line version of RNAsubopt with options "-ep 5 -p 1000 -noLP", which specify the percentage from the MFE (5%), random sample size (1000) and disable prediction of helices of length 1.

RNAfold was used to compute partition function and base-pair probabilities. RNAfold uses dynamic programming to compute the MFE secondary structure of a given RNA sequence, but when run with option '-p' it also computes base-pair probabilities.

Alifold was used to compute consensus secondary structure for *RPS17B *and *RPS6B *introns based on the alignment of introns in *Sensu stricto *species. It uses modified dynamic programming algorithms that add a covariance term to the standard energy model to compute a consensus secondary structure for a set of aligned RNAs.

RNAsubopt, RNAfold and Alifold are part of the Vienna RNA secondary structure package [22] (we used version 1.7). All four algorithms use free energy calculation based on Turner's nearest neighbour energy model [15,36-38].

### Distance calculation in an RNA secondary structure

Calculating the spatial distance between two nucleotides in a folded RNA molecule requires knowledge of the tertiary structure of the molecule. Since currently there are no reasonably reliable algorithms for predicting RNA tertiary structure, our distance calculation is based solely on RNA secondary structure. Considering that secondary structure is generally believed to play a crucial role in tertiary structure formation [34,35], this approach should give us a good approximation of the true spatial distance.

**Figure 10:**
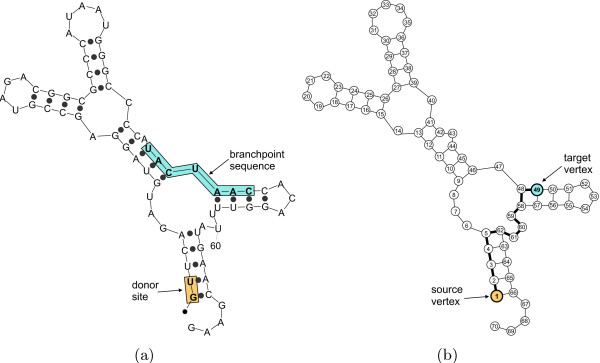
Conversion from the RNA secondary structure to the graph representing it. (a) Graphical representation of the secondary structure of an intron produced by mfold (filled-in circles represent base-pairing interactions, i.e., hydrogen bonds). (b) Graph representing the RNA structure in (a). The bolded path between the source and target vertices is the one found by the algorithm to be the shortest (*d*_*s *_= 11).

To calculate the structural branchpoint distance *d*_*s*_, we consider a predicted secondary structure of the intronic pre-mRNA as an undirected graph whose vertices are nucleotide bases and whose edges correspond to the bonds between the nucleotides. These bonds can be either sugar-phosphate bonds between the nucleotides in the RNA chain or the hydrogen bonds between paired bases in a given RNA secondary structure. Figure 10 shows the conversion from an RNA secondary structure to the secondary structure graph representing it. To compute the distance between two vertices in the graph, we employed Dijkstra's shortest-path algorithm [39]. Since Dijkstra's algorithm requires a directed graph, we represent each non-directed edge (*u*, *v*) as two directed edges, (*u*, *v*) and (*v*, *u*). All edges in the RNA secondary structure graph have uniform weight *w*(*u*, *v*) = 1.

In our implementation of the algorithm, the inputs to the program are a pseudoknot-free RNA secondary structure in dot-bracket notation (Vienna format) and the locations of two bases for which the distance needs to be calculated. These bases are the first nucleotide of the intron and the bulging A in the branchpoint sequence (UACUAAC). The output of the program is the shortest distance between these two bases, which we consider as the structural branchpoint distance (*d*_*s*_) for the given intron secondary structure. The program is available at .

### Mutant sequences

We used two basic strategies for designing intron mutants with desired structural characteristics. To obtain mutants with long structural branchpoint distances we aimed to disrupt a zipper stem that was bringing the donor site and the branchpoint sequence close together in the wildtype intron. Conversely, for the mutants designed to have efficient splicing we aimed to stabilize the zipper stem found in the wildtype intron. With these strategies in mind, we used a combination of a trial-and-error approach and secondary structure designs computed by RNA Designer [40] to obtain mutant sequence with desired structural characteristics.

**Figure 11:**
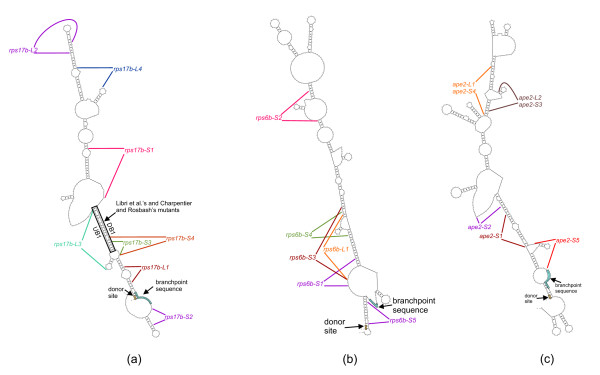
Location of mutations with respect to the secondary structure for (a) *RPS17B*, (b) *RPS6B*, and (c) *APE2 *introns. The two lines for each mutant indicate the beginning and end of the sequence segment that was modified.

Most of the intron mutants that we designed have segment substitutions around 20–30 nt long. Sequence segments of this size allowed us to rearrange the secondary structure of a mutant in a desired way. The exception is mutant *rps6b-S5 *which has three short insertions (8 nt in total) in the polypyrimidine tract of *RPS6B *intron. Mutant *rps17b-L3 *is a result of two 3-nucleotide-segment substitutions in Libri et al.'s [8] mutant *8mUB1 *(the middle sequence of lower case letters represents the original *8mUB1 *mutation). Similarly, mutant *rps17b-S3 *is a result of a 4-nucleotide-segment substitution in the *3mDB1 *mutant (the first segment of lower case letters represents the original *3mDB1 *mutation). Table 8 gives the location and sequence of mutant substitutions and Figure 11 depicts mutant locations with respect to the secondary structure of the introns we studied.

**Table 8:** Specifications for the new intron mutants used in our study.

**mutant**	**segment location**	**original sequence**	**substitution/insertion**
***rps17b-L1***	258–268 (11 nt)	UGAAGAGAGGU	augagacaacu
***rps17b-L2***	138–157 (20 nt)	GAUUAGAAAACUCCAUUACU	cuuaaguuaguaaauaccuc
***rps17b-L3***	22–47 (26 nt)	UGAAGCCGGAUAUGAUGGACUGGGC	uuaAGCCGcuacuacuUGGACUGucg
***rps17b-L4***	167–189 (23 nt)	AGAAGAGCGCUCAAUGAAGUAGU	uggcuuggguuaguaggugccuc
***rps17b-S1***	217–231 (15 nt)	AAUUGCUUUCGAAUG	uuucauguguucagc
***rps17b-S2***	280–286 (7 nt)	UAAGUUG	uacguac
***rps17b-S3***	246–253 (8 nt)	AUCCAAUG	uagCggcu
***rps17b-S4***	244–253 (10 nt)	UUAUCCAAUG	cuucaucaac
***rps6b-L1***	21–54 (34 nt)	CCUUAGAAUUCUAAUGAAUCAGCACGCGCUAACC	guauuuugggugugucccuguuauaaauaauacc
***rps6b-S1***	19–29 (11 nt)	AUCCUUAGAAU	uuuguuaguaa
***rps6b-S2***	87–113 (27 nt)	CACAAAUUAGUGCACUAUAAUAAAAAU	uuauaaauagugauaccauuugguaaa
***rps6b-S3***	21–57 (37 nt)	CCUUAGAAUUCUAAUGAAUCAGCACGCGCUAACCGGC	aaauuccaacguuucccugcaacaugccuuucuuccg
***rps6b-S4***	38–55 (18 nt)	AUCAGCACGCGCUAACCG	auucccaacagacugucc
***rps6b-S5***	337–345	GUAUUAUUU	GgUguucAUUAUUacaU
***ape2-L1***	159–175 (17 nt)	UGUUACCCUCAUAUUCU	ggguacaauuaauagag
***ape2-L2***	237–252 (16 nt)	GCAAUAGCUUAGGUAA	ccuucguacuuuuggg
***ape2-S1***	23–37 (15 nt)	CAAAGAAACAAGGAA	agggcagaaauagaa
***ape2-S2***	43–57 (15 nt)	AUACAUAAUAUAAAU	aacugguagguacgu
***ape2-S3***	237–252 (16 nt)	GCAAUAGCUUAGGUAA	caaugaaugagaacuc
***ape2-S4***	159–175 (17 nt)	UGUUACCCUCAUAUUCU	aaauauuaccuaagcua
***ape2-S5***	300–322 (23 nt)	CUCGUUACCGACCUUUGAGUUCU	uuaagcuuuuguguuugagaaca

The upper case letters represent the original sequences and the lower case letters represent substitution or insertion sequences. The first base of an intron is numbered 1.

### Generation and assaying of intron mutants

Using the *TRP1 *gene as a selectable marker, *RPS17B*, *RPS6B *and *APE2 *were tagged at their genomic locus with a -13MYC fragment to generate C-terminal protein fusions in yeast strains derived from a s288c background [41]. Western blotting with a MYC antibody (Covance Research Products) confirmed expression of the correct size protein product in each strain. The intron of the selected gene plus 5' and 3' flanking sequences were deleted through homologous recombination with the *URA3 *selectable marker in each of these tagged strains. Intron DNA containing sequences homologous to regions 5' and 3' of the *URA3 *insertion plus the selected intron mutations were created by PCR. Transformation of these fragments into the appropriate intron deletion strain results in recombination, removal of the *URA3 *gene, and insertion of the mutant intron sequence. The *URA3 *gene product leads to cell death when placed on 5-fluoroorotic acid (5-FOA) due to the conversion of 5-FOA to a toxic by product [42]. After transformation, cells can be selected on 5-FOA for those strains that have lost *URA3 *via insertion of the mutant intron, and thus can grow in the presence of 5-FOA. PCR was used to confirm that 5-FOA resistant strains were the result of insertion of the mutated intron in place of *URA3*. Each intron mutation was subsequently confirmed by sequencing. Strains containing the correct intron mutations were mated with a strain carrying a 13 MYC epitope tagged protein of a different molecular weight as an internal control and assayed for protein expression levels by western blotting. Western blotting was performed using 20–200 ng of whole cell lysate with a MYC antibody (Covance Research Products) and was quantitated after being developed with ECL Plus Western Blotting Detection reagent (Amersham Bioscience) using a Storm Imaging system (Amersham Bioscience). For each mutant assayed, the internal control was used to normalize protein loading, and the experiments were performed a minimum of 2 times on two independently derived mutant isolates.

### Yeast intron dataset

In order to obtain a high quality yeast intron dataset we consulted three databases: the Ares lab Yeast Intron Database [43], the Yeast Intron DataBase [44], and the Comprehensive Yeast Genome Database [45]. For additional information, we used the *Saccharomyces *Genome Database (SGD) [46]. We constructed our dataset by including introns that have consistent annotations between at least two of the three databases. We considered only introns from single-intron genes (which represent the majority of intron-containing genes in *S. Cerevisiae*) that interrupt the gene's coding region (this excluded introns found in the 5' UTR region). The number of introns found to have a consistent annotation between at least two databases was 214 (there are ~240 introns in the yeast genome). Eleven of these were excluded because they were not supported by the latest comparative genomic study [23], which labeled them as possible misannotations. The final dataset contains 203 yeast introns, 155 of which are experimentally verified and 48 are putative introns. There are 98 long (5'L) and 105 short (5'S) introns. We call this dataset the STRuctural INtron (STRIN) dataset. The STRIN dataset is available at .

## Authors' contributions

SR conceived the study, performed computational experiments and drafted the manuscript. HHH and AKM participated in the design and coordination of the study and together with BFO supervised the research project. BM designed and performed laboratory experiments and helped draft the manuscript. PH helped design laboratory experiments. All authors analyzed the results and reviewed drafts of the manuscript. All authors read and approved the final manuscript.

## Supplementary Material

Additional file 1 Minimum free energy structures for Libri et al.'s [8] mutants predicted by mfold. Click here for file

Additional file 2 Distribution histograms of structural branchpoint distances for (a) wt, (b) *UB1i*, (c) *DB1i*, (d) *UB1iDB1i*, (e) *mut-5*, (f) *mut-12*, and (g) *mut-18 *introns. Click here for file

Additional file 3 Structural characteristics of newly designed *RPS17B *mutants based on mfold predictions: ***d*_*s *_**– structural branchpoint distances for MFE and all suboptimal predictions within 5% from the MFE; **avg **– average ***d*_*s*_**; **bp prob **– base-pairing probability of interaction between the donor site and the branchpoint sequence based on the partition function. Click here for file

Additional file 4 Distribution histograms of structural branchpoint distances for (a) *rps17b-L1*, (b) *rps17b-L2*, (c) *rps17b-L3*, (d) *rps17b-L4*, (e) *rps17b-S1*, (f) *rps17b-S2*, (g) *rps17b-S3*, and (h) *rps17b-S4 *mutants. Click here for file

Additional file 5 Distribution histograms of structural branchpoint distances for (a) *RPS6B *wildtype intron, (b) *rps6b-L1*, (c) *rps6b-S1*, (d) *rps6b-S2*, (e) *rps6b-S3*, (f) *rps6b-S4*, and (g) *rps6b-S5 *mutants. Click here for file

Additional file 6 Distribution histograms of structural branchpoint distances for (a) *APE2 *wildtype intron, (b) *ape2-L1*, (c) *ape2-L2*, (d) *ape2-S1*, (e) *ape2-S2*, (f) *ape2-S3*, (g) *ape2-S4*, and (h) *ape2-S5 *mutants. Click here for file
