# Supplementary figures and images for: Correlation between the secondary structure of pre-mRNA introns and the efficiency of splicing in Saccharomyces cerevisiae
Source: BMC Genomics. 2008 Jul 29;9:355. doi: 10.1186/1471-2164-9-355 (PMC2536676; doi:10.1186/1471-2164-9-355)

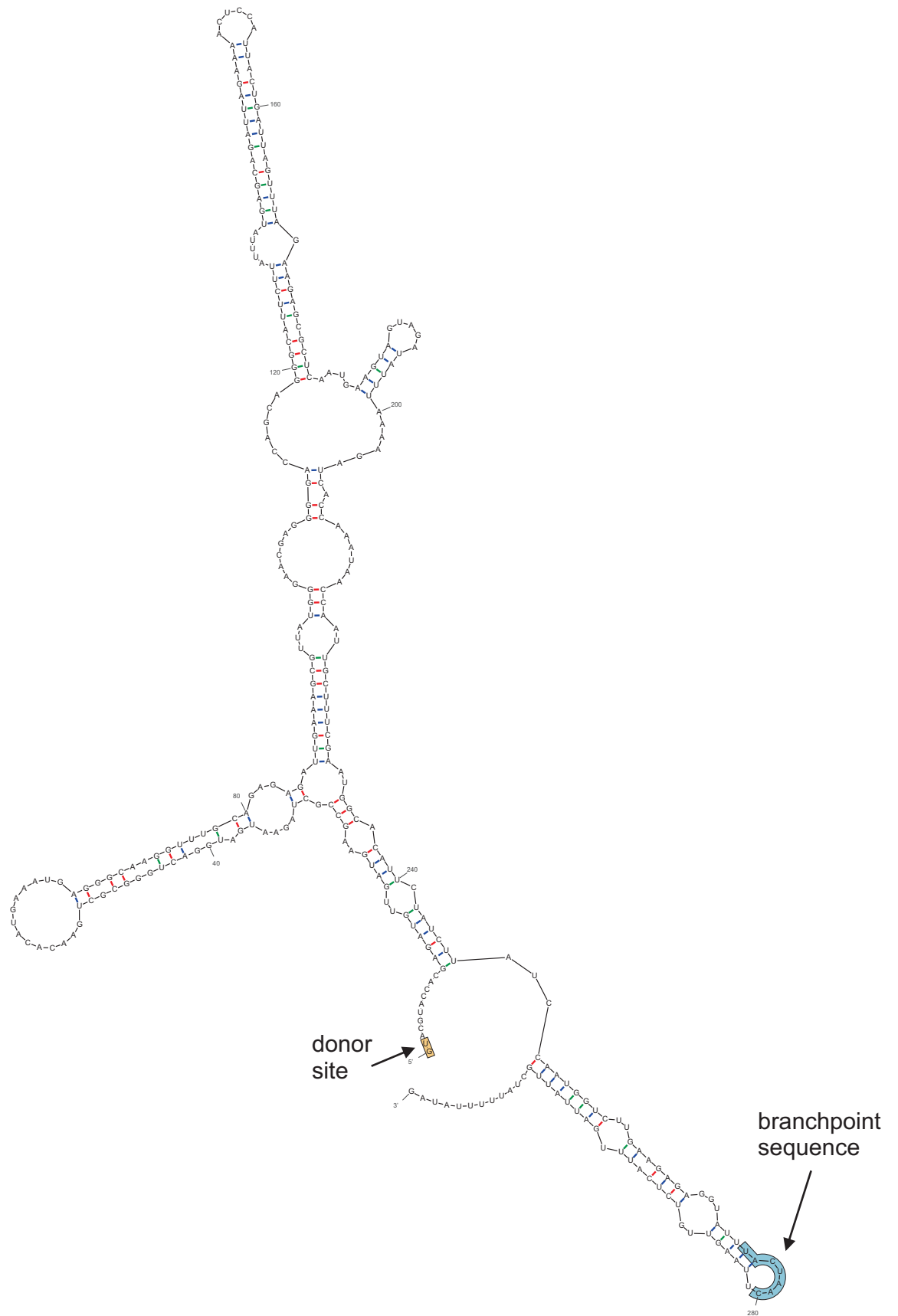

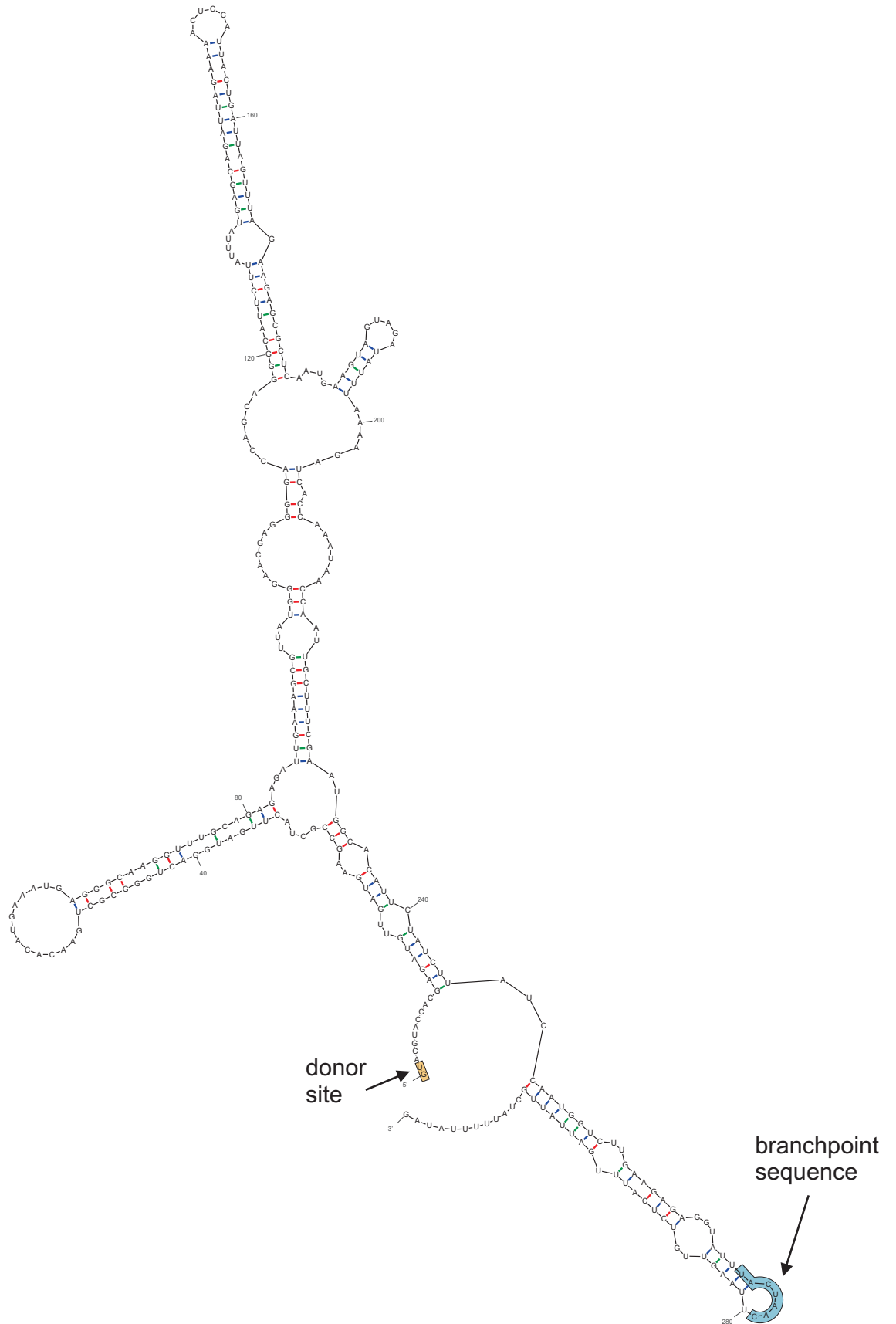



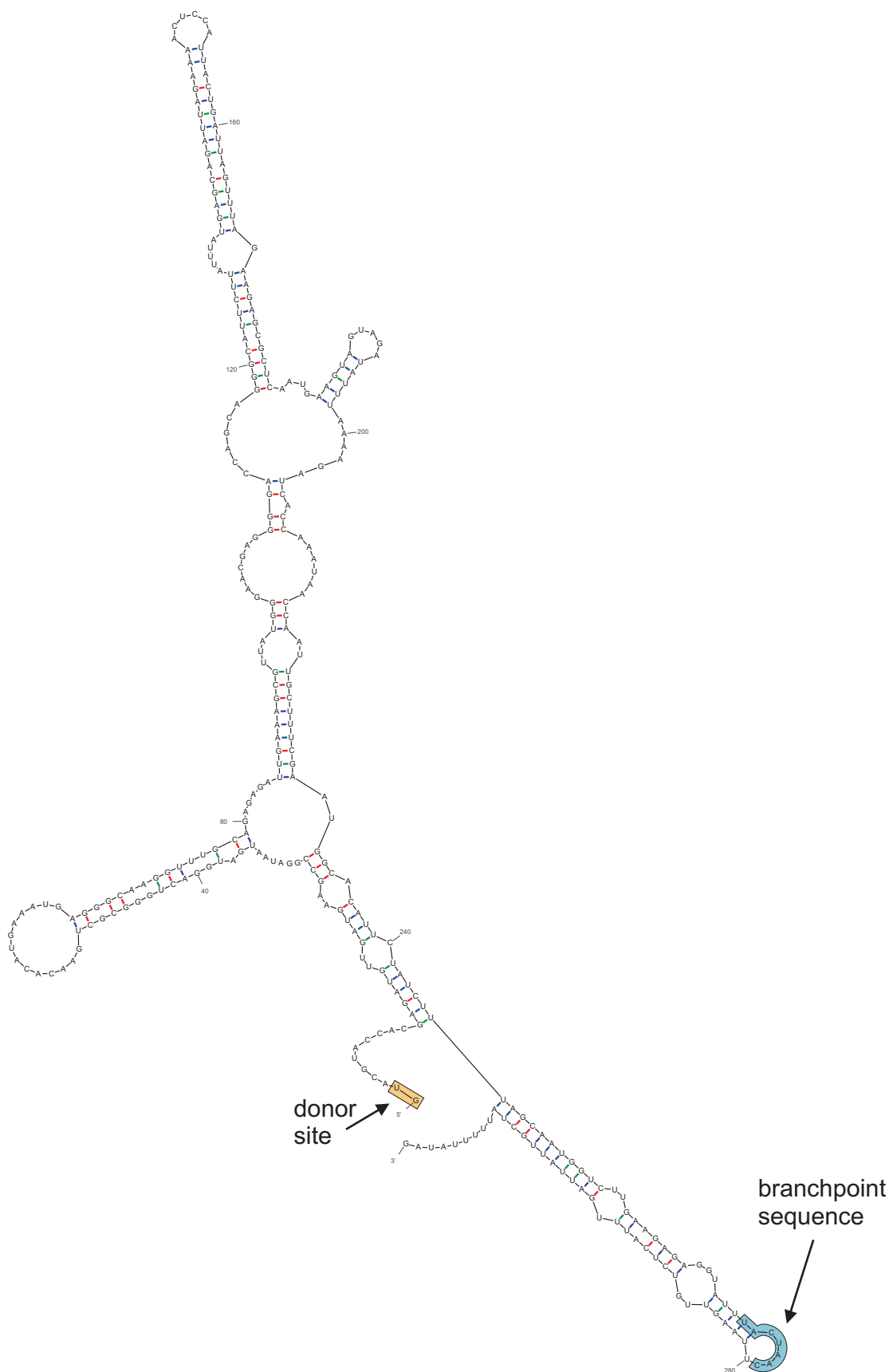

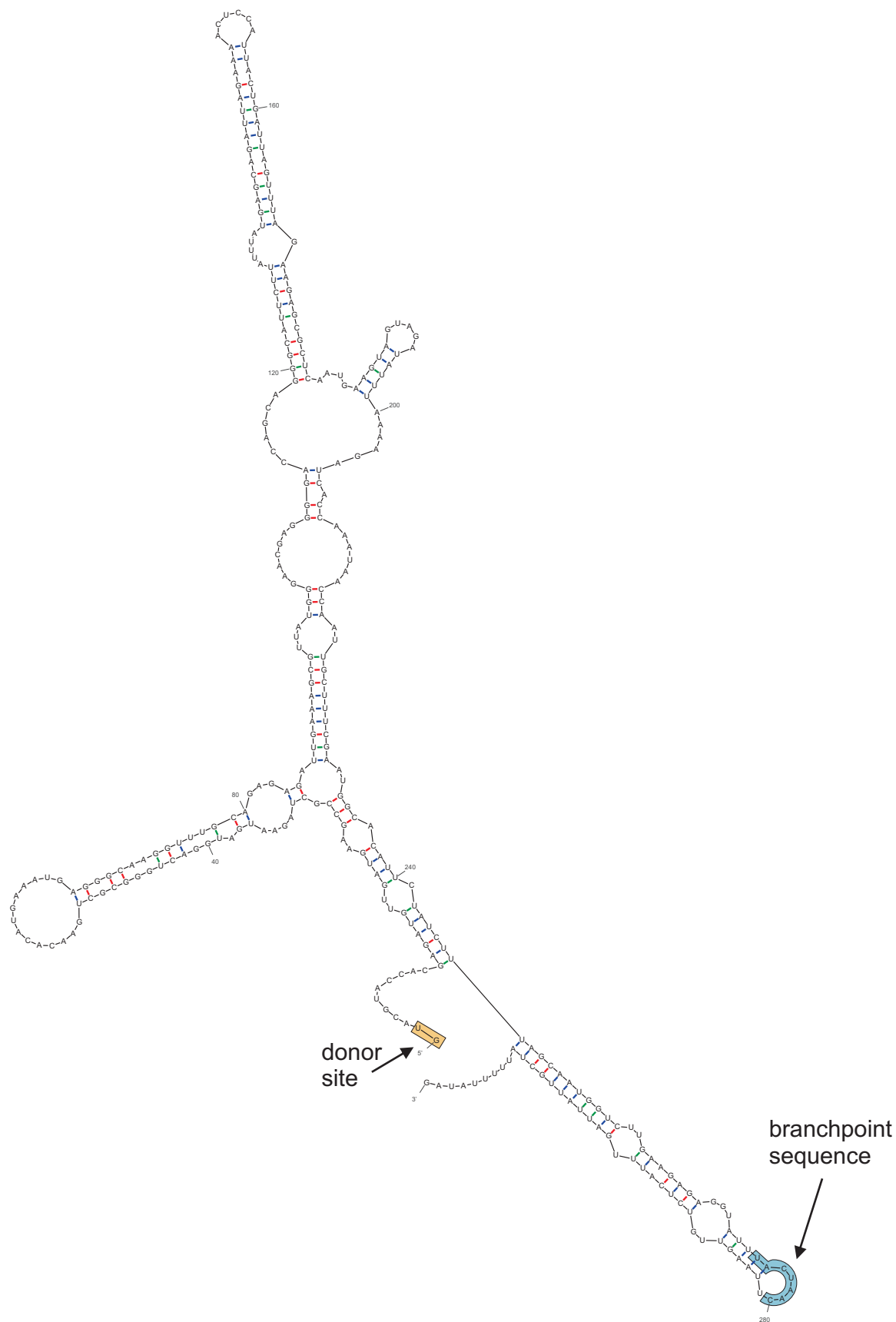

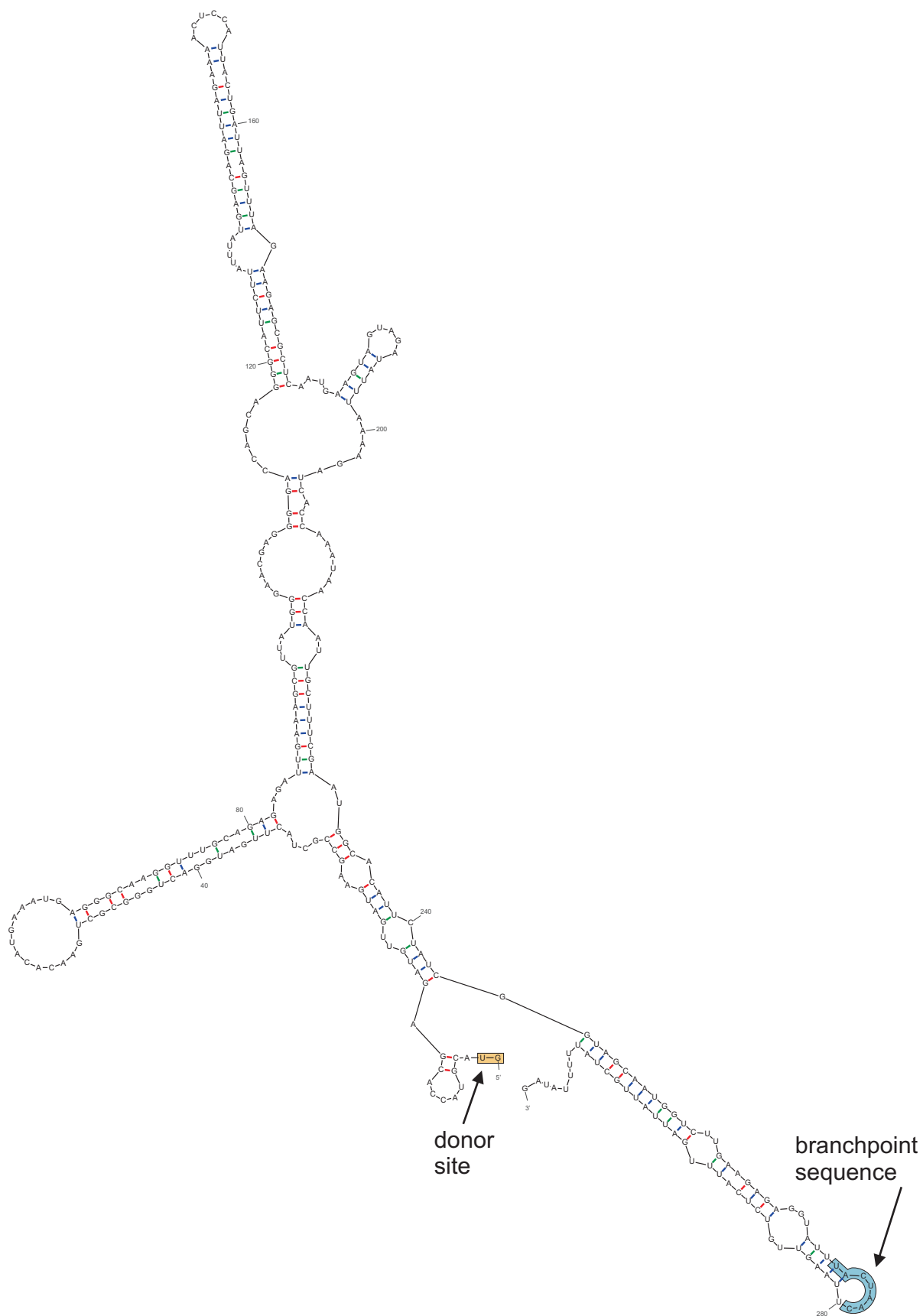

5mUB1\_5mDB1

Supplement: Additional file 1 — Minimum free energy structures for Libri et al.'s [8] mutants predicted by mfold. [file 1471-2164-9-355-S1.pdf]

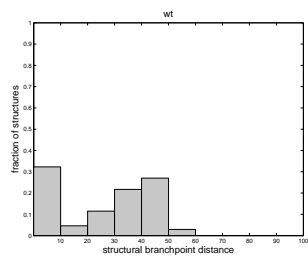

(a)

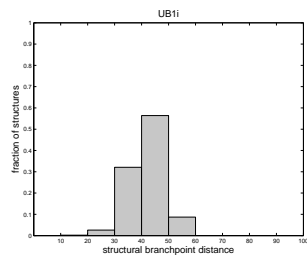

(b)

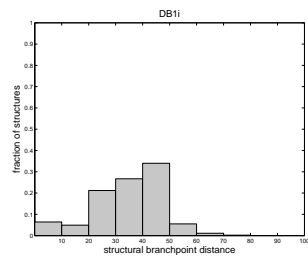

(c)

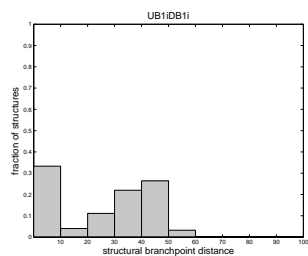

(d)

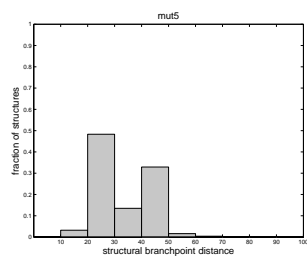

(e)

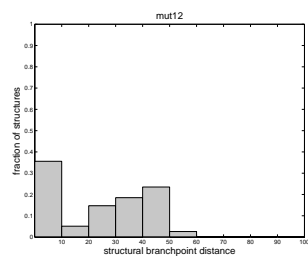

(f)

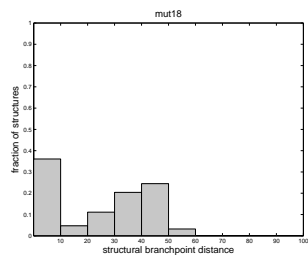

(g)

Supplement: Additional file 2 — Distribution histograms of structural branchpoint distances for (a) wt, (b) UB1i, (c) DB1i, (d) UB1iDB1i, (e) mut-5, (f) mut-12, and (g) mut-18 introns. [file 1471-2164-9-355-S2.pdf]

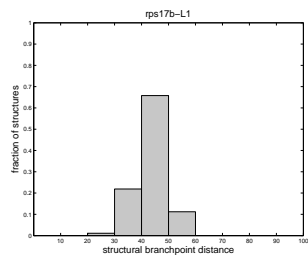

(a)

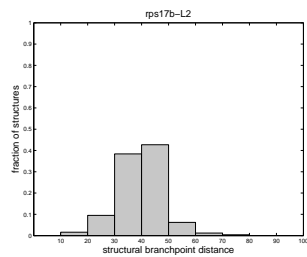

(b)

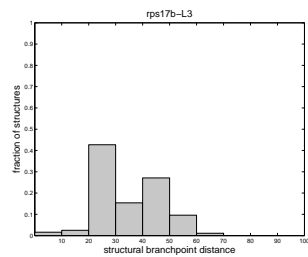

(c)

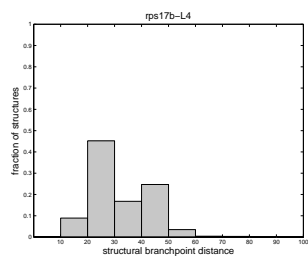

(d)

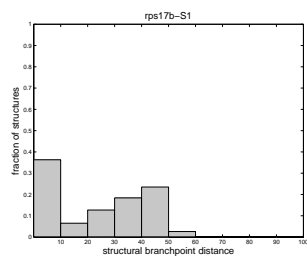

(e)

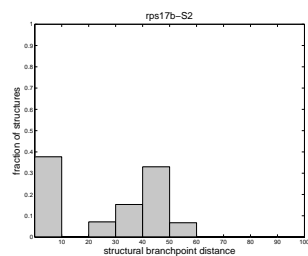

(f)

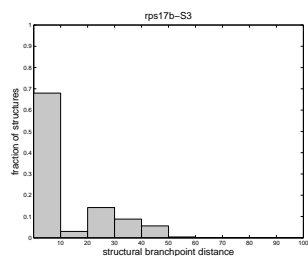

(g)

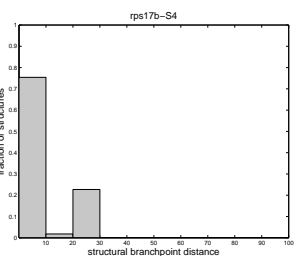

(h)

Supplement: Additional file 4 — Distribution histograms of structural branchpoint distances for (a) rps17b-L1, (b) rps17b-L2, (c) rps17b-L3, (d) rps17b-L4, (e) rps17b-S1, (f) rps17b-S2, (g) rps17b-S3, and (h) rps17b-S4 mutants. [file 1471-2164-9-355-S4.pdf]

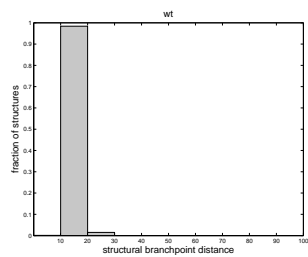

(a)

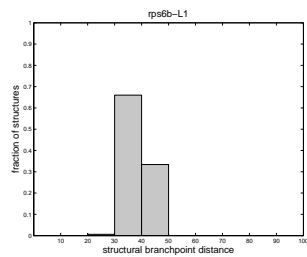

(b)

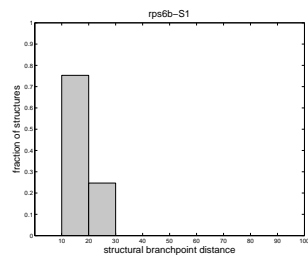

(c)

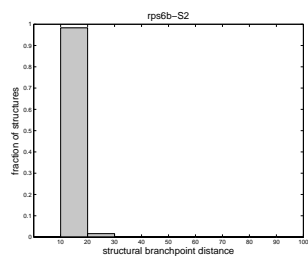

(d)

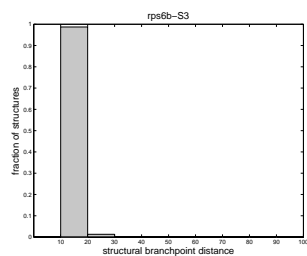

(e)

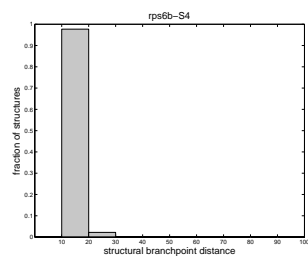

(f)

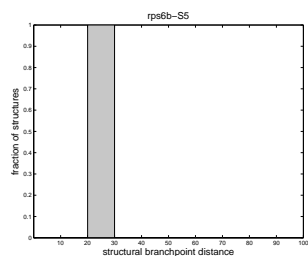

(g)

Supplement: Additional file 5 — Distribution histograms of structural branchpoint distances for (a) RPS6B wildtype intron, (b) rps6b-L1, (c) rps6b-S1, (d) rps6b-S2, (e) rps6b-S3, (f) rps6b-S4, and (g) rps6b-S5 mutants. [file 1471-2164-9-355-S5.pdf]

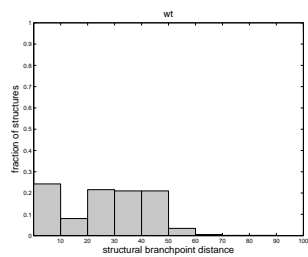

(a)

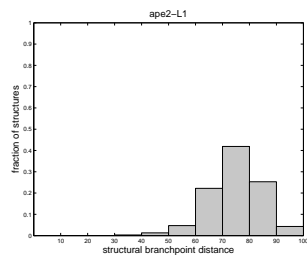

(b)

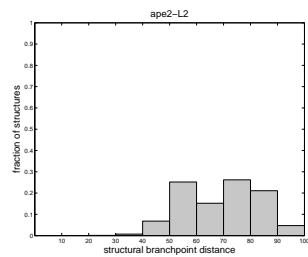

(c)

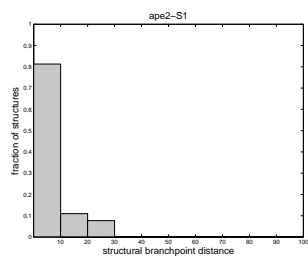

(d)

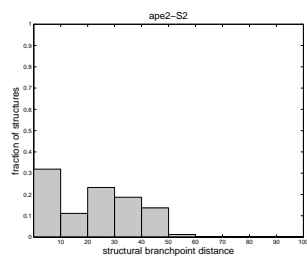

(e)

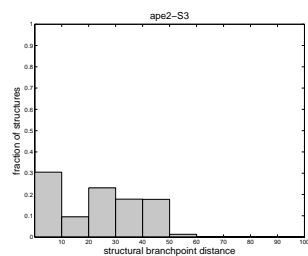

(f)

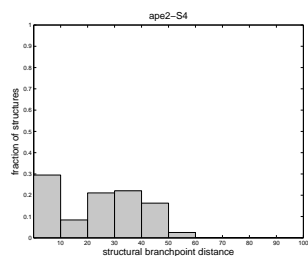

(g)

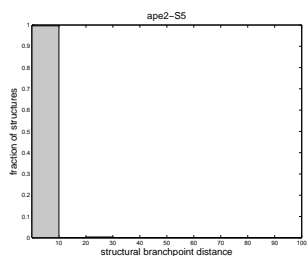

(h)

Supplement: Additional file 6 — Distribution histograms of structural branchpoint distances for (a) APE2 wildtype intron, (b) ape2-L1, (c) ape2-L2, (d) ape2-S1, (e) ape2-S2, (f) ape2-S3, (g) ape2-S4, and (h) ape2-S5 mutants. [file 1471-2164-9-355-S6.pdf]
