# Supplementary material for: Correlation between the secondary structure of pre-mRNA introns and the efficiency of splicing in Saccharomyces cerevisiae
Source: BMC Genomics. 2008 Jul 29;9:355. doi: 10.1186/1471-2164-9-355 (PMC2536676; doi:10.1186/1471-2164-9-355)
Supplement: Additional file 3 — Structural characteristics of newly designed RPS17B mutants based on mfold predictions: ds – structural branchpoint distances for MFE and all suboptimal predictions within 5% from the MFE; avg – average ds; bp prob – base-pairing probability of interaction between the donor site and the branchpoint sequence based on the partition function. [file 1471-2164-9-355-S3.pdf]

mutant	d_s	avg	bp prob
**wt**	**4**,28,**4**,42,43,**4**,**4**,44,**4**	21	0.40
rps17b-L1	41,45,45,45,41,45,41	43	0.0
rps17b-L2	45,45,49,49,45,44,45,25,48,45	44	0.0
rps17b-L3	21,54,21,54,42,25,42	37	0.21
rps17b-L4	25,25,44,49,25,25,49,49	36	0.04
rps17b-S1	**4**,**4**,28,45,**4**,**4**,18,**4**,**4**	13	0.40
rps17b-S2	10,28,10,42,10,10,10	17	0.03
rps17b-S3	**4**,28,42,18,21,44,**4**,**4**,**4**,**4**	17	0.80
rps17b-S4	**4**,**4**,**4**,**4**,28,**4**,**4**,**4**,**4**	7	0.70
